# Quantum chemical insight into the effects of the local electron environment on T_2_*-based MRI

**DOI:** 10.1038/s41598-021-00305-7

**Published:** 2021-10-21

**Authors:** M. S. Petronek, J. J. St-Aubin, C. Y. Lee, D. R. Spitz, E. G. Gillan, B. G. Allen, V. A. Magnotta

**Affiliations:** 1 Department of Radiation Oncology, University of Iowa, Iowa City, IA USA; 2 Department of Radiology, University of Iowa, Iowa City, IA USA; 3 Department of Chemistry, University of Iowa, Iowa City, IA USA; 4 Division of Free Radical and Radiation Biology, Department of Radiation Oncology, The University of Iowa Hospitals and Clinics, Iowa City, IA 52242-1181 USA; 5 Department of Radiology, The University of Iowa Hospitals and Clinics, Iowa City, IA 52242 USA

**Keywords:** Biophysical chemistry, Predictive markers

## Abstract

T_2_* relaxation is an intrinsic magnetic resonance imaging (MRI) parameter that is sensitive to local magnetic field inhomogeneities created by the deposition of endogenous paramagnetic material (e.g. iron). Recent studies suggest that T_2_* mapping is sensitive to iron oxidation state. In this study, we evaluate the spin state-dependence of T_2_* relaxation using T_2_* mapping. We experimentally tested this physical principle using a series of phantom experiments showing that T_2_* relaxation times are directly proportional to the spin magnetic moment of different transition metals along with their associated magnetic susceptibility. We previously showed that T_2_* relaxation time can detect the oxidation of Fe^2+^. In this paper, we demonstrate that T_2_* relaxation times are significantly longer for the diamagnetic, d^10^ metal Ga^3+^, compared to the paramagnetic, d^5^ metal Fe^3+^. We also show in a cell culture model that cells supplemented with Ga^3+^ (S = 0) have a significantly longer relaxation time compared to cells supplemented with Fe^3+^ (S = 5/2). These data support the hypothesis that dipole–dipole interactions between protons and electrons are driven by the strength of the electron spin magnetic moment in the surrounding environment giving rise to T_2_* relaxation.

## Introduction

T_2_* mapping is a clinically accepted imaging tool for the assessment of iron overload in the heart and liver^1–7^. In both organs, T_2_* relaxation times have a direct, inverse correlation with total iron content^8–11^. Recent studies suggest that T_2_* relaxation times also correlate with iron oxidation state. In an ex vivo model system, the addition of a reducing agent significantly increased T_2_* relaxation times^12^. Moreover, as part of an ongoing phase II clinical trial, glioblastoma patients treated with a combination of radiation, temozolomide, and pharmacological ascorbate demonstrated increased T_2_* relaxation times following pharmacological ascorbate treatment^13^. In this context, the authors propose that ascorbate can act as a reducing agent facilitating the conversion of ferric (Fe^3+^) to ferrous (Fe^2+^) iron and that T_2_* relaxation time is sensitive to iron oxidation state, in addition to total iron content.

T_1_, T_2_, and T_2_* relaxation are intrinsic contrast mechanisms in MR imaging. Following the application of a radiofrequency (RF) pulse at the resonant (i.e. Larmor) frequency, a portion of the protons will be tipped away from the Z-axis and into the XY-plane. Once the RF pulse is turned off, affected protons will begin to (1) re-align in Z-axis and (2) dephase in the XY-plane due to interactions with other protons. The time for affected proton spins to re-align in the Z-axis is the T_1_ relaxation time and the time for the same protons to dephase in the XY-plane is known as the T_2_ relaxation time. T_1_ and T_2_ relaxation are inherent properties of the tissue and does not include local field variations. T_2_* relaxation is the increased dephasing of proton spins in the XY-plane^14^ due to local magnetic field inhomogeneities from (a) intrinsic inhomogeneities associated with scanner variations and (b) deposition of endogenous or exogenous paramagnetic materials within the anatomical region of interest (e.g. iron). Protons in the presence of these magnetic field inhomogeneities dephase more rapidly resulting in the T_2_* relaxation times being shorter than T_2_ relaxation times.

Transition metals can exist in a variety of oxidation states with a wide array of spin configurations. The most physiologically relevant transition metal is iron, which can exist in the ferric (Fe^3+^) and ferrous (Fe^2+^) state, although it may also transiently exist as Fe^4+^^15–17^. Other transition metals are also present within tissues. Manganese (Mn) and copper (Cu) are both present in proteins such as superoxide dismutase 1 and 2 (SOD1 = CuZnSOD; SOD2 = MnSOD)^18,19^. Typically, the 5 d-orbitals of transition metals are degenerate in a labile state. However, when coordinated by a ligand (e.g. under physiological conditions), the d-orbitals exhibit a loss of degeneracy. The number of bound ligands and ligand bond strengths determine the associated d-orbital splitting. This loss of degeneracy gives rise to an angular momentum associated with unpaired electrons for these metals. For example, a high spin iron complex with an octahedral ligand configuration has a quantum spin (S) of S = 5/2 (Fe^3+^) or 2 (Fe^2+^). Conversely, low spin iron will have S = 1/2 (Fe^3+^) or 0 (Fe^2+^). Based on the number of d-orbital electrons, transition metals exhibit a wide array of quantum spins (Supplemental Table S1). The unpaired electrons thus exhibit a spin magnetic moment (${\mu }_{s}$) that is related to the number of unpaired electrons (Eq. (1)):

1 $${\mu }_{s}=g\sqrt{S(S+1)},$$

where *g* is the electronic g-factor (≈ 2.002).

The spin magnetic moment of transition metals is directly related to their measured paramagnetic molar susceptibility (${\chi }_{mol}$) (Eq. (2)):

2 $${\mu }_{s}= \sqrt{\frac{3k{\chi }_{mol}T}{N{\beta }^{2}}}=2.828\sqrt{{\chi }_{mol}T},$$

where $k$ = Boltzmann’s constant, N is Avogadro’s number, β is the Bohr magneton, and T is the temperature in Kelvin (K). The unpaired electron’s orbital angular momentum contribution to transition metal ion magnetic moments is usually small or negligible, in contrast to lanthanide ions where this can be a significant additional magnetic contribution. Thus, ${\mu }_{s}$ or S is often sufficient to estimate ${\chi }_{mol}$ for transition metal ions.

To evaluate the relationship between the number of unpaired electrons and magnetic susceptibility through the associated magnetic moment, Eqs. (1) and (2) can be rearranged to show:

3 $${\chi }_{mol}=\frac{S(S+1)}{1.99T}.$$

A paramagnetic compound has a non-zero electron spin and associated magnetic moment that can engage in proton-electron dipole–dipole interactions. Thus, we hypothesize that the electron spin magnetic moment has a significant impact on T_2_* relaxation and that T_2_* mapping is sensitive to alterations in the electronic properties of biologically relevant transition metals.

## Results

### T_2_* relaxation times are directly proportional to magnetic susceptibility

**Figure 1:**
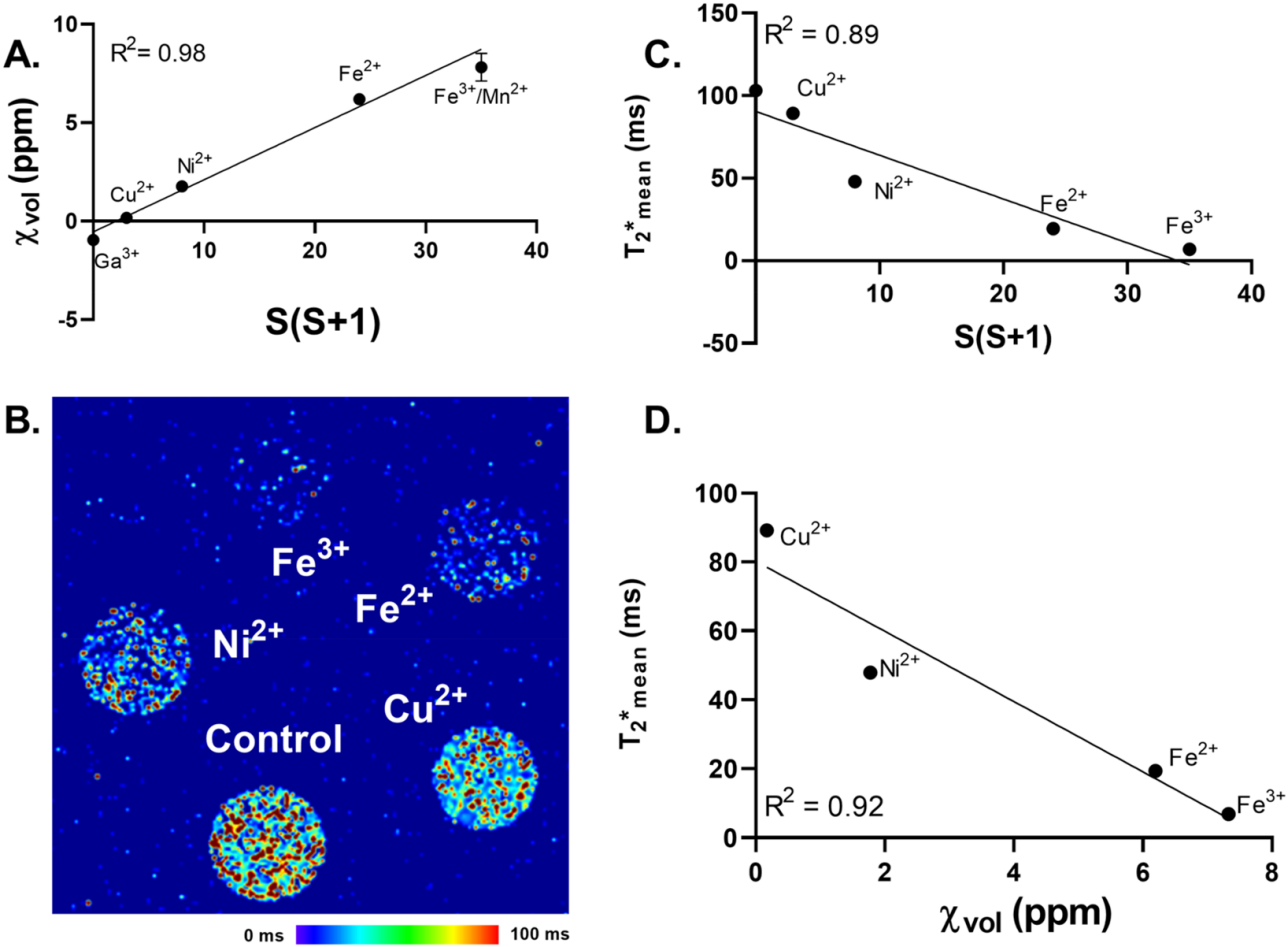
T_2_* and χ_vol_ is directly proportional to the number of unpaired electrons. Volumetric magnetic susceptibility measures were taken of 1 M concentrations of Ga(NO_3_)_3_ (Ga^3+^), CuSO_4_ (Cu^2+^), NiCl_2_ (Ni^2+^), (NH_4_)_2_Fe(SO_4_)_2_·6H_2_O (FAS, Fe^2+^), Fe(NO_3_)_3_ (Fe^2+^), and MnSO_4_ (Mn^2+^) in 4 mm O.D. quartz EPR tubes. Samples were prepared at room temperature (25 °C). (**A**) Linear correlation between experimentally determined volumetric magnetic susceptibility and the spin-only magnetic moment of various inorganic species. Each metal was left unchelated and diluted in double-distilled H_2_O at a 1 M concentration. (**B**) T_2_* map of transition metals (100 µM) embedded in a 1% agarose gel and scanned using a 7 T scanner. (**C**) Linear correlation between experimentally determined T_2_* relaxation times and the spin-only magnetic moment of various inorganic species. (**D**) Linear correlation between T_2_* relaxation times and volumetric magnetic susceptibilities determined experimentally. Each experiment was done with triplicate measures.

Equation (3) indicates that ${\upchi }_{\mathrm{mol}}$ is directly proportional to the spin quantum number associated with the number of unpaired electrons. We reviewed previously published data regarding molar susceptibility of various transition metal compounds (Fe^3+^, Mn^2+^, Fe^2+^, Ni^2+^,Cu^2+^; Supplemental Table S2) and found that their relative ${\upchi }_{\mathrm{mol}}$ values are directly proportional to the associated S(S + 1) term (R^2^ = 0.73, r = 0.98; Supplemental Fig. S1). To test the sensitivity of T_2_* relaxation to the electron configuration, we first measured the volumetric magnetic susceptibility (χ_vol_) of various transition metals at an equal concentration (1 M). We observed that χ_vol_ is linearly dependent upon the number of quantum spin (S(S + 1)) of the transition metal (R^2^ = 0.98; Fig. 1A). To validate this result, we compared the known values for different metal complexes (${\upchi }_{\mathrm{mol},\mathrm{ exp}})$ to our experimentally determined susceptibility values ${(\upchi }_{\mathrm{vol},\mathrm{ obs}})$ and found a linear relationship as anticipated (R^2^ = 0.99, Supplemental Fig. S2). Our experimental ${\chi }_{vol}$ measures are reported in ppm, which can be used to calculate the associated ${\chi }_{mol}$ values using the following relationship (Eq. (4)):

4 $${\upchi }_{\mathrm{mol}}=\frac{\mathrm{M}}{\uprho }*{\upchi }_{\mathrm{vol}},$$

where M is the molar mass (kg mol^−1^) and ρ (m^3^ kg^−1^).

To test if these paramagnetic metals have a similar effect on T_2_*; Cu^2+^, Ni^2+^, Fe^2+^, and Fe^3+^ were embedded in a 1% agarose gel at an equal concentration (100 µM) and scanned using a 7 T magnet to generate T_2_* maps (Fig. 1B). T_2_* relaxation times were linearly dependent on the quantum spin (S(S + 1)) for the examined transition metals (R^2^ = 0.89, Fig. 1C). T_2_* relaxation times were also inversely proportional to the experimentally determined ${\upchi }_{\mathrm{vol}}$ measurements (R^2^ = 0.89, r =  − 0.96; Fig. 1D). However, it is worth noting that the signal will be nearly fully decayed for material with very large ${\chi }_{vol}$ (e.g. paramagnetic MR contrast), making the evaluation of signal changes difficult. These data suggest that T_2_* relaxation times may be significantly affected by the electron spin magnetic moment (µ_s_) and number of unpaired electrons (S) in the surrounding environment.

### T_2_* mapping detects Fenton chemistry

To determine if the oxidation of Fe^2+^ via Fenton chemistry reaction (Eq. (5)) is reflected in T_2_* relaxation, H_2_O_2_ was incubated at room temperature and pressure with ferrous ammonium sulfate (FAS; Fe^2+^).

5 $${\mathrm{H}}_{2}{\mathrm{O}}_{2}+ {\mathrm{Fe}}^{2+} \to {\mathrm{Fe}}^{3+}+ {\mathrm{HO}}^{-}+ {\mathrm{HO}}^{\cdot }.$$

**Figure 2:**
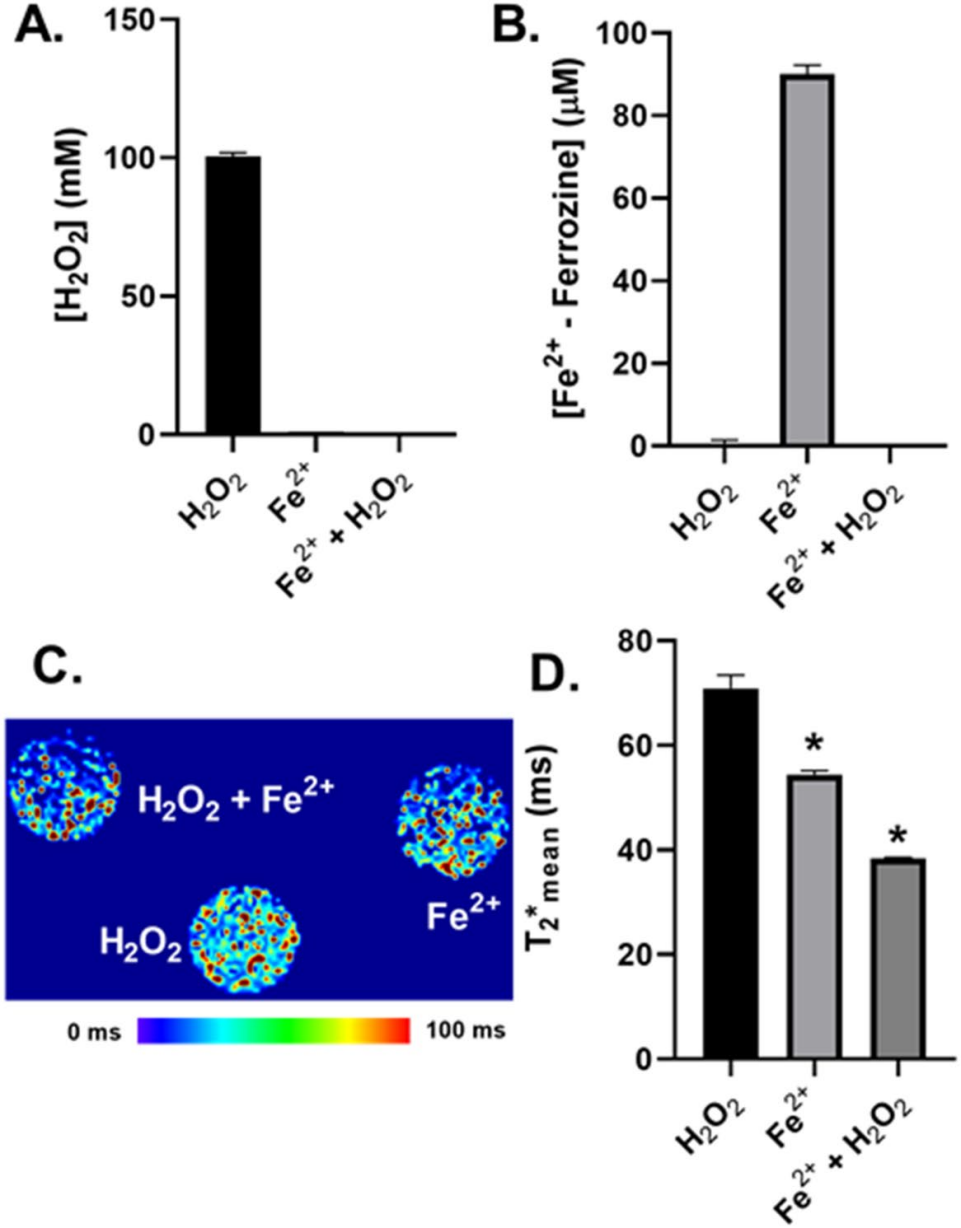
T_2_^*^ mapping detects Fenton chemistry. (**A**) Measurement of H_2_O_2_ using UV–Vis spectroscopy (240 nm; ε = 46.3 mol^−1^ cm^−1^; 1 cm pathlength) following 15 min incubation of 1.5 mM FAS ((NH_4_)_2_Fe(SO_4_)_2_ 6H_2_O) with 100 mM H_2_O_2_ for 15 min in double-distilled H_2_O. (**B**) Measurement of Fe^2+^ following 1:15 dilution in 5 mM ferrozine using UV–Vis spectroscopy (562 nm; ε = 27,900 mol^−1^ cm^−1^; 1 cm pathlength). (**C**) Representative T_2_^*^ map of 1:15 dilution in 1% agarose gel. (**D**) Mean T_2_^*^ relaxation times quantified using Slicer3D software by generation a 1 mm ROI for each phantom and calculating the mean T_2_^*^ relaxation time using the data quantification package within the software. Error bars represent SD of n = 3 biological replicates. Statistical analysis was performed using a one-way ANOVA test with statistical significance defined as a false positivity rate less than 5% (*p < 0.05).

Following a 15 min incubiation, the H_2_O_2_ concentration was 100.4 ± 1.4 mM as compared to 0.59 ± 0.02 mM with FAS (Fig. 2A). To assess the oxidation of Fe^2+^ by H_2_O_2_, each solution (H_2_O_2_, FAS, and H_2_O_2_ + FAS) was diluted 1:15 in 5 mM ferrozine buffer and measured using UV–Vis spectroscopy at 562 nm (Supplemental Fig. S3). This technique allows for the detection of a purple ferrozine-Fe^2+^ complex, while ferrozine-Fe^3+^ remains colorless^20^. Following H_2_O_2_ addition, Fe^2+^ concentration was 0.37 ± 0.01 µM compared to a final, unperturbed Fe^2+^ concentration of 90.0 ± 1.2 µM (Fig. 2B). Each sample (H_2_O_2_, FAS, and H_2_O_2_ + FAS) was then diluted 1:15 in 1% agarose gel and scanned at 7 T to generate a T_2_* map (Fig. 2C). Quantitative analysis of each phantom revealed a mean T_2_* relaxation time of 70.9 ± 1.5 ms for the H_2_O_2_ sample, 54.4 ± 0.4 ms for the unperturbed Fe^2+^ sample, and 38.4 ± 0.1 ms following oxidation (Fig. 2D). These data suggest that T_2_* mapping can detect the chemical conversion of Fe^2+^ to Fe^3+^ via Fenton chemistry.

### T_2_* relaxation differentiates between Ga^3+^ and Fe^3+^

**Figure 3:**
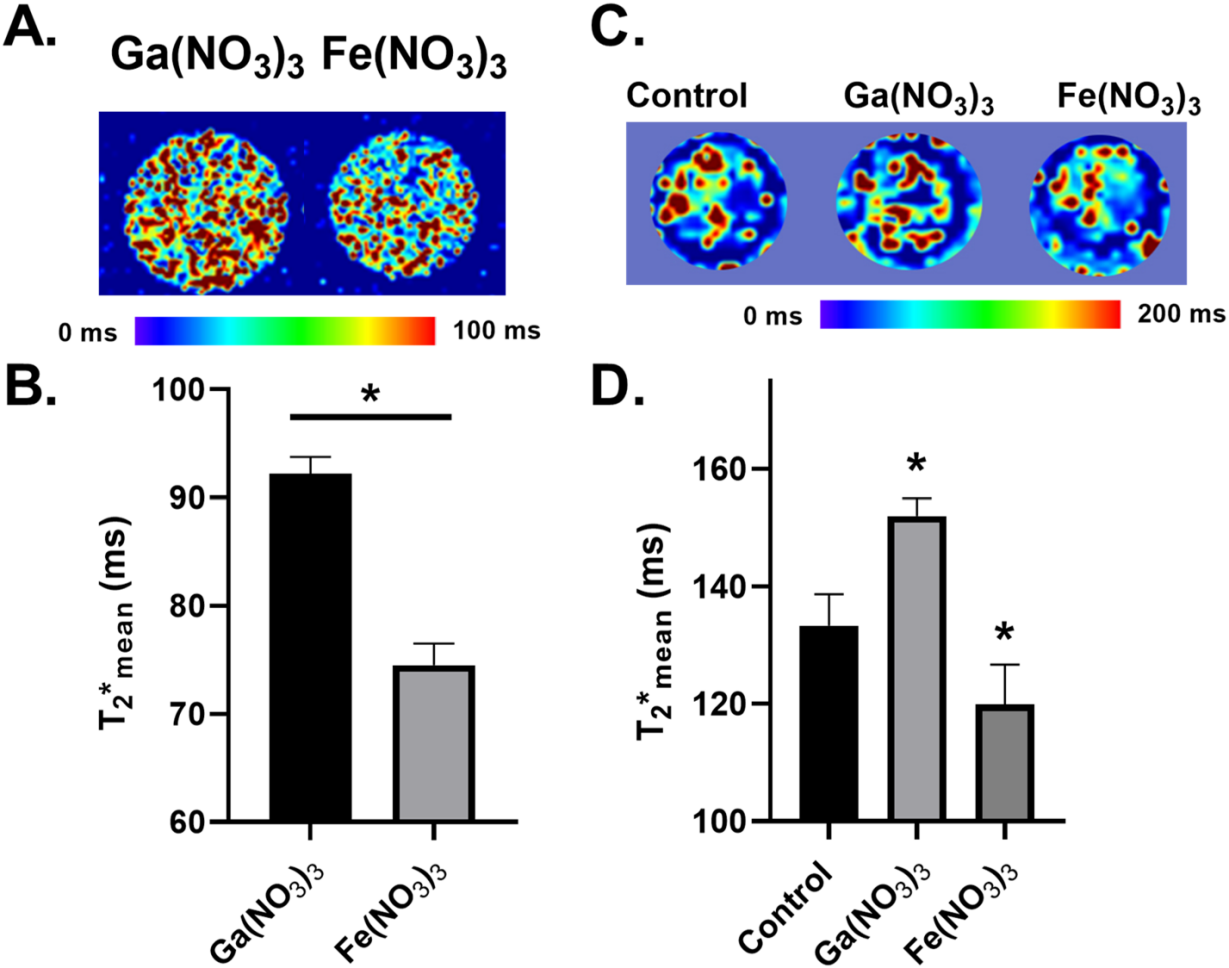
T2* relaxation differentiates between Ga^3+^ and Fe^3+^. (**A**) 100 µM Ga(NO_3_)_3_ and Fe(NO_3_)_3_ solutions embedded in a 1% agarose gel in 4 mm O.D. quartz EPR tubes and scanned using a 7 T magnet to generate T_2_* maps. (**B**) Mean T_2_* relaxation times quantified using Slicer3D software by generating a 1 mm ROI for each phantom and calculating the mean T_2_* relaxation time using the data quantification package within the software. *p < 0.05 using a paired t-test. Error bars represent SEM of three replicates. (**C**) Representative T_2_* map of U251 glioblastoma cell pellets following a 3 h treatment of 120 µM Ga(NO_3_)_3_ or Fe(NO_3_)_3_. (**D**) Mean T_2_* relaxation times quantified using Slicer3D software by generating a 1 mm ROI for each phantom and calculating the mean T_2_* relaxation time using the data quantification package within the software. Error bars represent SD of n = 3 biological replicates. *p < 0.05 using a one-way ANOVA test.

Ga^3+^ is a d^10^ post-transition metal that is diamagnetic due to a completely full d orbital set with ten paired electrons (S = 0) while Fe^3+^ is a highly paramagnetic d^5^ metal with S = 5/2 because of its five unpaired electrons. As expected, the Ga^3+^ phantom had a significantly longer T_2_* relaxation time (92.2 ± 1.6 ms) compared to Fe^3+^ (74.5 ± 2.0 ms) (Fig. 3A,B).

Ga^3+^ and Fe^3+^ also behave similarly in biological systems as they both bind transferrin in circulation and enter cells through transferrin-receptor mediated endocytosis^21–23^. Treatment of U251 glioblastoma cells with either 120 µM Ga^3+^ or Fe^3+^ for 3 h caused a significant increase in T_2_* relaxation times (152.0 ± 1.7 ms) in Ga^3+^ treated cells compared to untreated cells (133.3 ± 3.1 ms) while Fe^3+^ resulted in a significant decrease in T_2_* relaxation (119.9 ± 3.9 ms; Fig. 3C,D).

## Discussion and conclusions

Several studies have showed that T_2_* mapping is a useful tool for assessing iron concentrations in vivo. T_2_* is widely accepted as an imaging approach to assess for iron overload in the heart and liver^5–11^. It has also been shown that T_2_* is directly proportional to iron concentration in various cortical brain regions^24^. However, more recent studies have suggested that T2* relaxation can detect tissue iron-oxidation state^12,13^. We believe this is because electron contributions from the local environment are driving changes in T_2_* due to the different d orbital valence configurations of Fe^3+^ (d^5^) and Fe^2+^ (d^4^). In an ex vivo model, human cadaveric brain tissue was exposed to a reductant to convert Fe^3+^ to Fe^2+^ or iron extraction to reduce the iron content of the tissue^12^. In both conditions, T_2_* was increased further supporting the iron concentration dependence (iron extraction) and spin-dependence (iron reduction) theories. In a study reporting the preliminary results of a phase II trial for glioblastoma patients treated with pharmacological ascorbate, a small cohort of patients showed a significant increase in T_2_* relaxation times 4 h after an 87.5 g infusion of ascorbate^13^. Ascorbate (vitamin C) is a one-electron reducing agent that readily converts Fe^3+^ to Fe^2+^ and can kill cancer cells in an iron-dependent mechanism^25–27^. This study also showed that Fe^3+^ has a much greater concentration-dependent effect on T_2_* relaxation than Fe^2+^, although Fe^2+^ still decreased T_2_*^13^. Taken together, this suggests that T_2_* relaxation is dependent on both the concentration and spin-configuration of the paramagnetic metal being considered.

In this study, we show that T_2_* relaxation times can detect transition metals that have varying electron spin contributions (µ_s_) and are linearly proportional to their associated magnetic susceptibility (χ_vol_). We show that T_2_* relaxation times are sensitive to Fe^2+^ oxidation and able to detect Fenton chemistry reactions. Reactions with H_2_O_2_ are thought to be a major contributor to Fe-mediated oxidative damage in cells^17,28^. Fenton chemistry is a critical oxidation step following the radiolysis of water and can lead to ionizing radiation induced DNA damage^28–30^. Similar to iron, copper, manganese, and nickel are also able to undergo Fenton-type reactions leading to DNA damage^31–34^. Therefore, being able to readily detect alterations in metal oxidation state may provide significant information regarding the biochemical processes that are central to metal catalyzed pathologic processes and therapeutic response (e.g. radiotherapy) We also demonstrate that T_2_* mapping is able to differentiate between diamagnetic (d^10^; S = 0) Ga^3+^ and paramagnetic (d^5^; S = 5/2) Fe^3+^ metals as the high magnetic susceptibility of Fe^3+^ has a significant shortening of the T_2_* relaxation time compared to Ga^3+^. We validated this effect in a relevant, controlled, in vitro model system that controlled for several important variables including: (1) metal concentration, (2) metal oxidation state, (3) cellular uptake. U251 glioblastoma cells were treated for 3 h with identical concentrations of either Ga^3+^ or Fe^3+^ (120 µM). Ga^3+^ and Fe^3+^ are both taken into cells via transferrin—receptor mediated endocytosis to control for metal uptake^23^. Therefore, by supplementing cells with two metals where the only notable variable is the electron spin contribution, we show that the addition of a diamagnetic metal (Ga^3+^) increases T_2_* relaxation times while the paramagnetic Fe^3+^ decreases T_2_* relaxation times. This suggests that T_2_* relaxation may be useful in detecting diamagnetic/paramagnetic shifts in cells or tissues (e.g. influx/efflux of O_2_ to detect hypoxia).

In conclusion, we provide a revised understanding that changes in T_2_* relaxation times are being altered by proton-electron dipole–dipole interactions. This novel interpretation may allow investigators and clinicians to utilize T_2_* mapping to probe not only metal concentrations, but also, metal spin states. This understanding may be employed to develop new, non-invasive biomarkers to better understand human pathology, therapeutic response, and drug delivery by probing electron motion and metal metabolic changes in vivo.

## Materials and methods

### Magnetic susceptibility measures

Magnetic susceptibility measurements were performed by dissolving the desired metal to a concentration of 1 M in double–distilled H_2_O. Metals used in this study are provided in supplemental information (Supplemental Table S1). The metal solution was then put in a 4 mm O.D. Wilmad—quartz EPR tube (4 mm O.D.; Wilmad-LabGlass, Vineland, NJ, 707-SQ-250 M), sealed with parafilm, and measured at room temperature using a Johnson**-**Matthey MSB Evans magnetic susceptibility balance. Measurements were done in comparison to a water blank.

### MRI studies

Phantoms were generated by dissolving the desired metal in double-distilled H_2_O to get an initial concentration of 1.5 mM. Appropriate metals were then diluted 1:15 in 1% agarose gel (Seaplaque low gelling temperature agarose; Sigma-Aldrich, A9414) and allowed to solidify in EPR tubes. Images were collected on a 7T GE MR901 small animal scanner, a part of the Small Animal Imaging Core at the University of Iowa. T_2_* weighted images were collected using a gradient echo sequence (TR = 10 ms, TE = 2.2, 8.2, 14.2, and 20.2 ms, 256 × 256 resolution, 2 signal averages). A B0 shimming routine was performed to mitigate the effect of macroscopic filed inhomogeneity on T_2_* measurements. The echo time (TE) is the time between the radiofrequency pulse and the sampling of the MRI signal, the repetition time (TR) is the time between two repeated pulse cycles, and B0 is the main external magnetic field strength (7T). T_2_* maps were generated using a combination of 4 echo times collected and fitting each voxel to a mono-exponential curve using in-house python code. Images were imported to Slicer3D software where regions of interest (ROIs) were delineated and mean T_2_* values were calculated using the label statistics tool within Slicer3D^35^.

### Ultraviolet–visible light spectroscopy

Ultraviolet–visible light (UV–Vis) spectroscopic measurements were performed using a Beckman DU600 spectrophotometer. Samples were diluted to appropriate concentrations in double-distilled H_2_O and placed in a 1 cm cuvette. Fe^2+^—ferrozine complex formation being measured using a 400–800 nm wavelength scan and H_2_O_2_ was monitored using the absorbance at 240 nm. Fe^2+^—ferrozine and H_2_O_2_ concentrations were calculated using Beer’s Law using the absorbance at 562 nm (ε_562_ = 27,900 mol^−1^ cm^−1^) and 240 nm (ε_240_ = 46.3 mol^−1^ cm^−1^), respectively.

### Cell cultures

U251 glioma cells were cultured in DMEM-F12 media (15% FBS, 1% penicillin-strep, 1% Na-pyruvate, 1.5% HEPES, 0.1% insulin, and 0.02% fibroblast growth factor). Cells were plated in 100 mm^2^ dishes and grown to 70–80% confluence at 21% O_2_. Cells were either left untreated or supplemented with 120 µM Ga (NO_3_)_3_ or Fe(NO_3_)_3_ for 3 h at 21% O_2_. Cells were trypsinized and harvested by spinning at 1200 RPM for 5 min. Supernatant media was aspirated and cells were resuspended in 250 µL DPBS. Cells were then placed in the appropriate well of a PCR plate embedded in 1% agarose gel. Cells were allowed to collect at the bottom of the well to form a dense pellet for approximately 30 min. Following pellet formation, the entire phantom containing all groups was scanned in the MR901 using a 6.0 cm FOV while all other parameters for the multi-echo gradient echo sequence was the same as used for the phantom study.

## Supplementary Information

Supplementary Information.
