# Supplementary material for: Quantum chemical insight into the effects of the local electron environment on T2*-based MRI
Source: Sci Rep. 2021 Oct 21;11:20817. doi: 10.1038/s41598-021-00305-7 (PMC8531323; doi:10.1038/s41598-021-00305-7)
Supplement: Supplementary file 1 — Supplementary Information. [file 41598_2021_305_MOESM1_ESM.docx]

**Supplemental Data for**

***Nature Scientific Reports***

**Quantum chemical insight into the effects of the local electron environment on T_2_*-based MRI**

**MS Petronek^1*^, JJ St – Aubin^1^, CY Lee^2^, DR Spitz^1^, EG Gillan^3^, BG Allen^1Ɨ^, VA Magnotta^2Ɨ*^**

^1^Department of Radiation Oncology, University of Iowa, Iowa City, USA

^2^Department of Radiology, University of Iowa, Iowa City, USA

^3^Department of Chemistry, University of Iowa, Iowa City, IA USA

**Contents**

**Page 2: Supplemental table 1. Transition metals with differing electronic spin properties used in the study.**

**Page 3: Supplemental table 2. Molar susceptibility of different Fe^2+^ and Fe^3+^ compounds.**

**Page 4: Supplemental figure 1. Correlation between known χ_mol_ values and transition metals with differing number of unpaired electrons.**

**Page 5: Supplemental figure 2.** **Correlation between observed χ_vol_ measures and known χ_mol_ values for transition metals with differing number of unpaired electrons.**

**Page 5: Supplemental figure 3. Representative UV-Vis spectra for Fe^2+^ - ferrozine complex formation.**

| **Supplemental table 1. Transition metals with differing electronic spin properties used in this study.** | | | | | |
| --- | --- | --- | --- | --- | --- |
| Transition metal Ion | Compound | | e^-^ configuration | ^b^spin quantum number (S) | ^a^μ_s_ |
| Fe^3+^ | Fe(NO_3_)_3_ | | d^5^ | ±5/2 | $\sqrt{35}$ |
| Fe^2+^ | (NH_4_)_2_Fe(SO_4_)_2_·6H_2_O | | d^6^ | ±2 | $\sqrt{24}$ |
| Mn^2+^ | MnSO_4_ | | d^5^ | ±5/2 | $\sqrt{35}$ |
| Ni^2+^ | NiCl_2_ | | d^8^ | ±1 | $\sqrt{8}$ |
| Ga^3+^ | Ga(NO_3_)_3_ | | d^10^ | 0 | 0 |
| Cu^2+^ | CuSO_4_ | | d^9^ | ±1/2 | $\sqrt{3}$ |
| ^a^$\mu_{s}= \sqrt{S(S+1)}g$  ^b^assuming high-spin properties in an aqueous solution | | | | | |
|  | |  | | | |

| **Supplemental table 2. Molar susceptibility of different transition metal compounds^a^.** | | | | |
| --- | --- | --- | --- | --- |
| Compound | Fe oxidation state | spin quantum number (S) | χ_mol_ (10^-6^ cm^3^ mol^-1^)^b^ | Avg. χ_mol_ (10^-6^ cm^3^ mol^-1^) |
| FeBr_2_ | Fe^2+^ | ±2 | +13600 | +10730 |
| FeCO_3_ |  |  | +11300 |  |
| FeCl_2_ |  |  | +14750 |  |
| FeCl_2_ * 4H_2_O |  |  | +12900 |  |
| FeF_2_ |  |  | +9500 |  |
| FeI_2_ |  |  | +13600 |  |
| FeO |  |  | +7200 |  |
| FeSO_4_ |  |  | +12400 |  |
| FeSO_4_ * H_2_O |  |  | +10500 |  |
| FeSO_4_ * 7H_2_O |  |  | +11200 |  |
| FeS |  |  | +1074 |  |
| FeCl_3_ | Fe^3+^ | ±5/2 | +13450 | +13096 |
| FeCl_3_ * 6H_2_O |  |  | +15200 |  |
| FeF_3_ |  |  | +13760 |  |
| FeF_3_ * 3H_2_O |  |  | +7870 |  |
| Fe(NO_3_)_3_ * 9H_2_O |  |  | +15200 |  |
| MnBr_2_ | Mn^2+^ | ±5/2 | +13900 | +13060 |
| MnCO_3_ |  |  | +11400 |  |
| MnCl_2_ |  |  | +14350 |  |
| MnF_2_ |  |  | +10700 |  |
| MnI_2_ |  |  | +14400 |  |
| MnSO_4_ |  |  | +13660 |  |
| NiBr_2_ | Ni^2+^ | ±1 | +5600 | +4407 |
| NiCl_2_ |  |  | +6145 |  |
| NiF_2_ |  |  | +2410 |  |
| NiI_2_ |  |  | +3875 |  |
| NiSO_4_ |  |  | +4005 |  |
| CuBr_2_ | Cu^2+^ | ±1/2 | +685 | +1154 |
| CuCl_2_ |  |  | +1080 |  |
| CuF_2_ |  |  | +1050 |  |
| Cu(NO_3_)_2_*6H_2_O |  |  | +1625 |  |
| CuSO_4_ |  |  | +1330 |  |
| ^a^Magnetic susceptibility of the elements and inorganic compounds. (Fermi National Accelerator Laboratory) accessed from: http://www.fizika.si/magnetism/MagSusceptibilities.pdf  ^b^molar susceptibility given in CGS-system units | | | | |


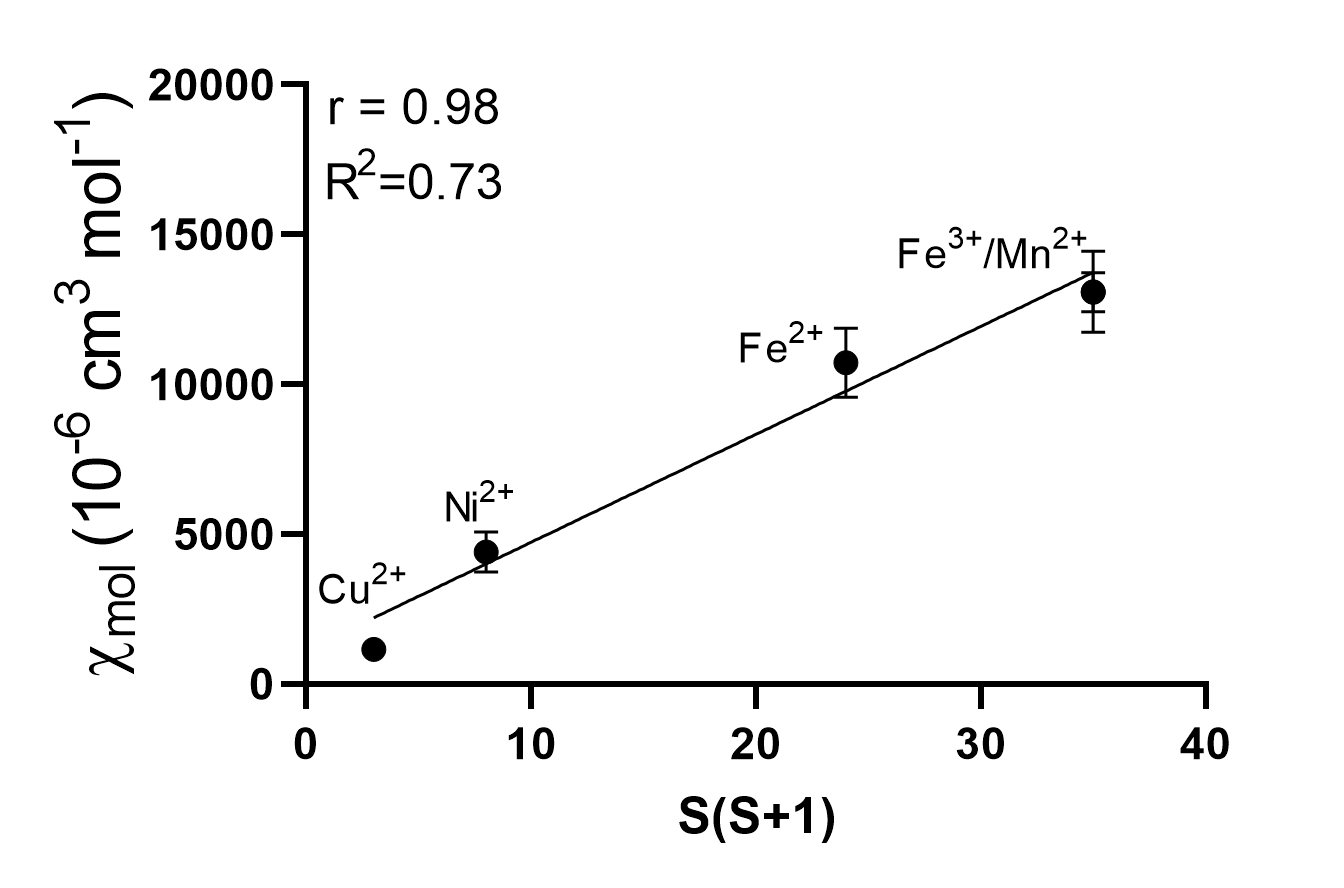


**Supplemental figure 1.** Correlation between known χ_mol_ values and transition metals with differing number of unpaired electrons. Known values acquired from *Magnetic susceptibility of the elements and inorganic compounds.* (Fermi National Accelerator Laboratory).


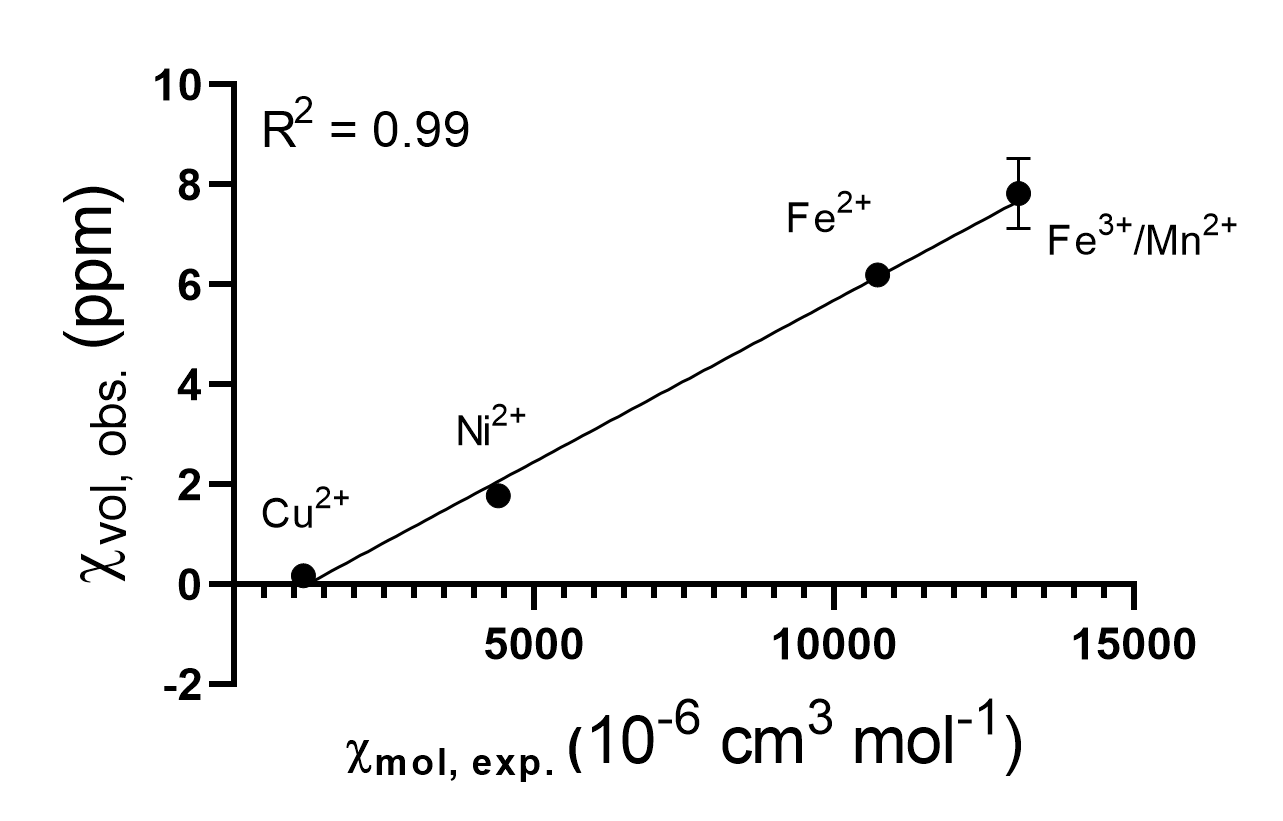


**Supplemental figure 2.** Correlation between observed χ_vol_ measures and known χ_mol_ values for transition metals with differing number of unpaired electrons. Known values acquired from *Magnetic susceptibility of the elements and inorganic compounds.* (Fermi National Accelerator Laboratory).


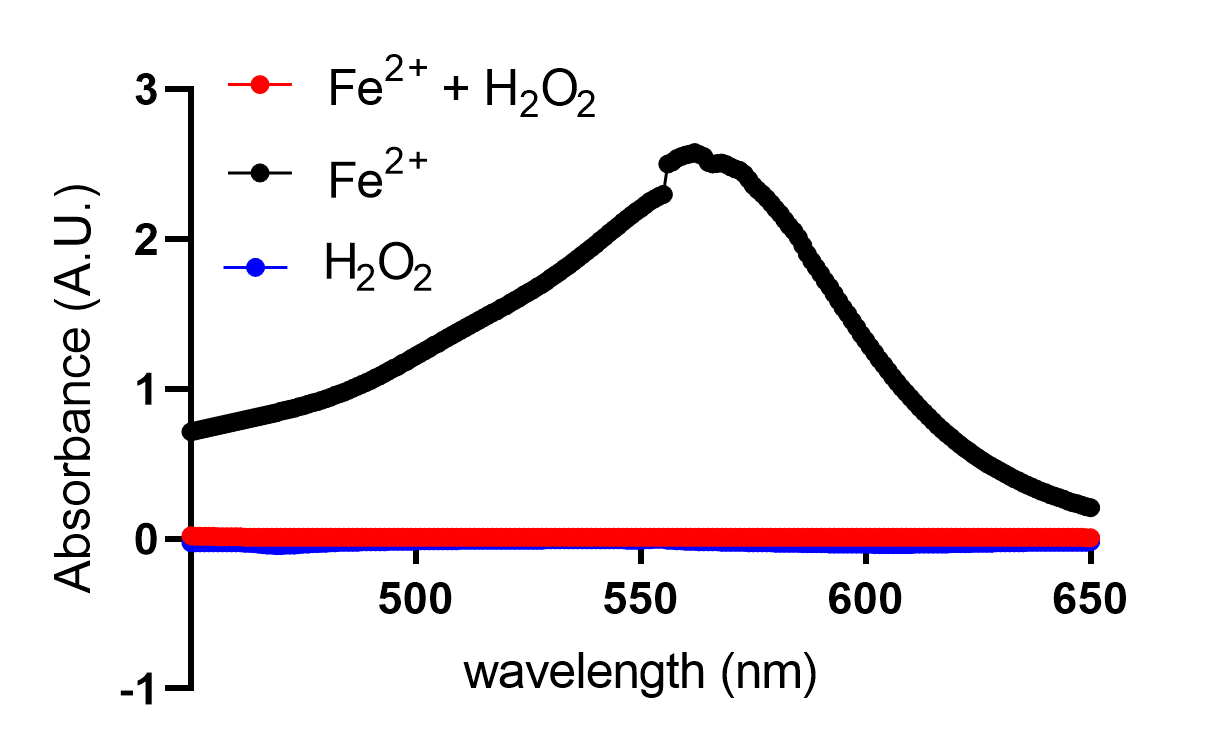


**Supplemental figure 3.** Representative UV-Vis spectra for Fe^2+^ - ferrozine complex formation. Fe^2+^ concentrations were calculated using Beer’s Law with ε_562_ = 27,900 mol^-1^ cm^-1^.
